# Bone Metastasis As the Initial Presentation of a Hidden Bronchogenic Carcinoma

**DOI:** 10.7759/cureus.28081

**Published:** 2022-08-16

**Authors:** Anas Ibraheem

**Affiliations:** 1 Internal Medicine, Imamein Kadhimein Medical City, Baghdad, IRQ

**Keywords:** bronchogenic carcinoma, chest x ray, diagnostic approach, advanced malignant tumour, oncology, palliative therapy, pathological fracture, bone metastasis, lung carcinoma

## Abstract

Primary malignant bone tumours are on the whole rare, while secondary bone tumours are much more common. Up to 40% of bone metastases are associated with lung cancer. This case report highlights a rare presentation of metastatic bone disease as the initial presentation of a primary lung malignancy and only very few cases were mentioned in literature with the same presentation of no clinical signs of the primary lung pathology, except for an unexpected radiological finding of a suspicious lung lesion.

An 85-year-old gentleman presented with a progressive lower backache radiating to both lower limbs over a period of 4 weeks associated with difficulty in walking, significant weight loss, and decreased appetite. A skeletal survey showed only spondylolisthesis. However, no clinical improvement was noticed with conventional therapy. Examination of the respiratory, gastrointestinal, and genitourinary systems was normal. Ultrasonography of the abdomen and pelvis, and the findings of the colonoscopy did not add anything. During the third week of follow-up, the patient reported unbearable severe pain in the left arm. A plain radiograph revealed a pathological fracture of the humerus. Secondary bone metastasis was suspected. Although the patient was a non-smoker and there were no clinical signs of underlying lung disease, a simple plain chest radiograph, unexpectedly, showed a suspicious right lower lobe lesion. Therefore, a contrast-enhanced computerized tomography (CT) scan for the chest, abdomen, and pelvis was done which revealed a right lower lobe lesion of bronchogenic carcinoma with distant metastasis. Unfortunately, the patient died after 3 weeks of palliative therapy when he was admitted to the hospital with acute renal failure and septic shock.

Bone metastases in lung cancer predict a poor prognosis and short-term survival. The diagnosis of such a challenging presentation requires a high index of suspicion. If the patient had been sent for a plain chest radiograph at first, lots of time and effort could be saved in reaching the diagnosis without the need for further sophisticated or invasive diagnostic procedures.

## Introduction

Primary malignant bone tumours are on the whole rare, while secondary bone tumours are much more common [1]. Up to 40% of bone metastases are associated with lung cancer [2]. The most common clinical symptom of bone metastasis is bone pain, which is usually localized and progresses slowly [3]. Lower backache is a common complaint for patients who present to physiotherapists and it is rarely associated with serious medical pathology (i.e. cancer) [4]. However, cancer as a cause can not be ruled out if the patient is older than 50 years old, exhibits unexplained weight loss, has a history of cancer, and does not respond to conservative intervention [5]. Numerous patients with multiple myeloma initially present with inexplicable backache, while 26%-34% of them would present with pathologic fracture [6,7]. On the other hand, advanced prostate cancer often develops bone metastases, leading to bone pain [8].

Lung cancer with bone metastasis is common [2], although metastatic bone disease as the initial presentation of primary lung carcinoma is rare, and only very few cases were mentioned in literature with the same presentation [9,10,11,12].

## Case presentation

**Figure 1:**
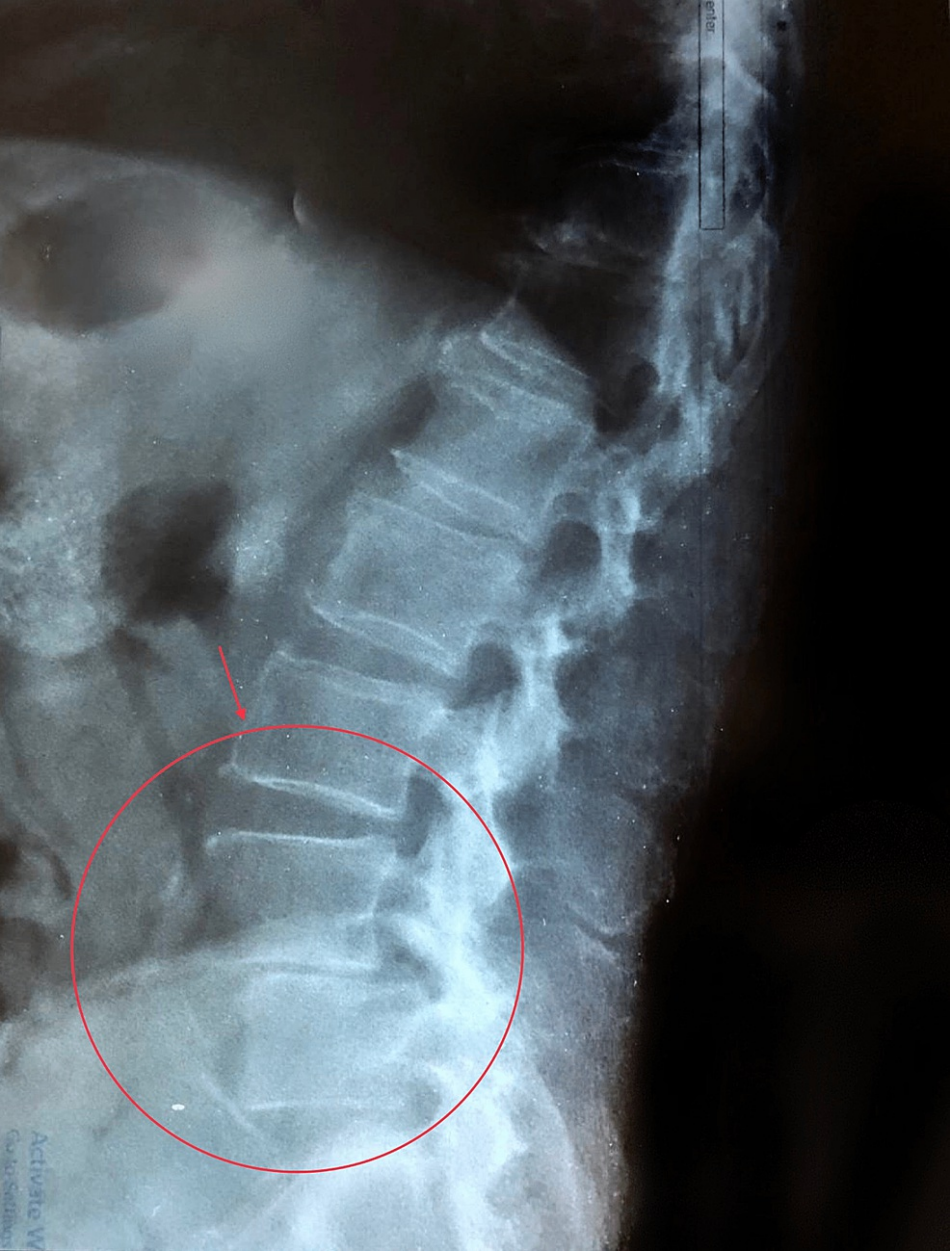
Plain lumbo-sacral radiograph showing L4-5 spondylolithesis and signs of osteoarthropathy.

In late April 2022, an 85-year-old gentleman presented with a progressive lower backache radiating to both lower limbs over a period of 4 weeks associated with difficulty in walking. He denied any recent trauma. A plain lumbo-sacral radiograph showed evidence of L4-5 spondylolisthesis and signs of osteoarthropathy (Figure 1). The general practitioner diagnosed him clinically with spondylolisthesis, but no improvement was noticed after 1 month of conventional therapy and analgesia.

He was referred to the medical consultation unit where he was reassessed. His pain was 8 on a pain scale from (0-10) where eight and above was considered severe pain. He complained of decreased appetite and significant unintended weight loss of 10 kg over the last 3 months with sleep disturbance. However, he didn’t have fever or nocturnal sweating. In the systemic review, he didn’t report any respiratory, gastrointestinal or genitourinary symptoms. He had a positive family history of different types of malignancy in the first degree relatives as following: his oldest brother died at the age of 65 from colorectal cancer after 5 years from diagnosis, his youngest brother has been diagnosed with colorectal cancer at age of 58 & he is still alive after a successful surgical resection, his youngest sister died at the age of 53 from advanced endometrial cancer.

He is a retired bank accountant with an insignificant past medical history or drug history. He was a non-smoker, but he consumed alcohol occasionally (approximately 2 glasses of whiskey per month).

On examination, he was a conscious, oriented, cachectic elderly male with pale conjunctiva. When his back was examined, no obvious deformity was seen. However, a severe tenderness over the lumber vertebrates associated with positive straight leg raise test for both lower limbs at 45 degrees of hip flexion was noticed. However, there was no neurological deficit.

There was nothing significant of the respiratory system, as both lungs were clear on auscultation and his SpO2 level was 97% on ambient air. Moreover, abdominal examination didn’t add anything as it was soft, non-tender with no organomegaly or free fluid. Both genital and digital rectal examination (DRE) were unremarkable. Furthermore, no lymphadenopathy was noticed.

On investigation, his complete blood count showed a haemoglobin of 10.6 g/dL and raised white blood cells (13.3 × 10^9^/L) with normal differentiation. However, his platelet count was normal. A peripheral blood film showed normocytic normochromic red blood cells and leukocytosis with left shift, but no blast cells were identified. ESR was 90 mm/hour and CRP was 14 mg/L.

**Figure 2:**
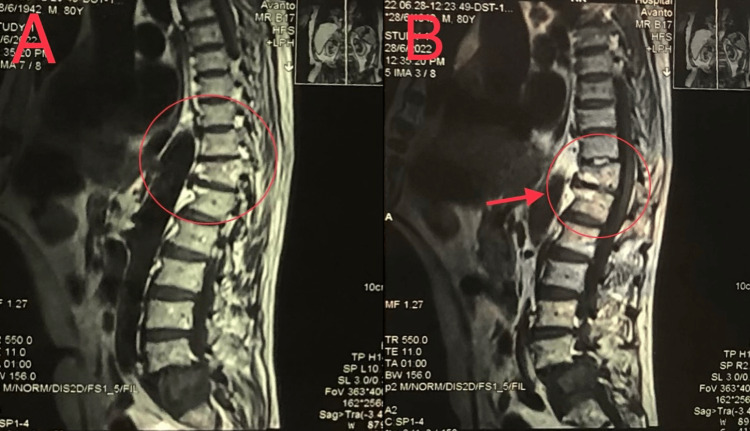
(A) and (B) images showing different MRI parasagittal sections of the spine with myelography representing abnormal signal intensity with anterior wedge compression in D11 and D12.

A magnetic resonance imaging (MRI) was done with myelography for the lumbo-sacral spine which revealed an abnormal signal intensity with anterior wedge compression in D11 and D12 (Figure 2).

The overall presentation of unexplained lower backache with evidence of anaemia and very high ESR level in an old age patient, raised the suspicion towards multiple myloma (MM) as the provisional diagnosis. However, the normal renal function test, normal electrolyte levels including ionised calcium level (1.3 mmol/L), and lack of abnormalities on the serum protein and urine protein electrophoresis made MM less likely to be the primary malignancy in this case. The diagnosis became much more challenging, especially, when the patient and his family refused bone marrow aspiration and biopsy.

Parathyroid hormone level was (38 pg/mL). Alkaline phosphatase was mildly elevated (160 IU/L), while the other liver enzymes were normal. Tumour markers were unremarkable including PSA (3.4 ng/mL), CEA (1.8 ng/mL), alpha fetoprotein (7.5 ng/mL) and CA19-9 (2 U/mL). Ultrasonography for the abdomen and pelvis didn’t reveal any abnormality. Also, colonoscopy was entirely normal despite the high risk family history of colorectal cancer.

**Figure 3:**
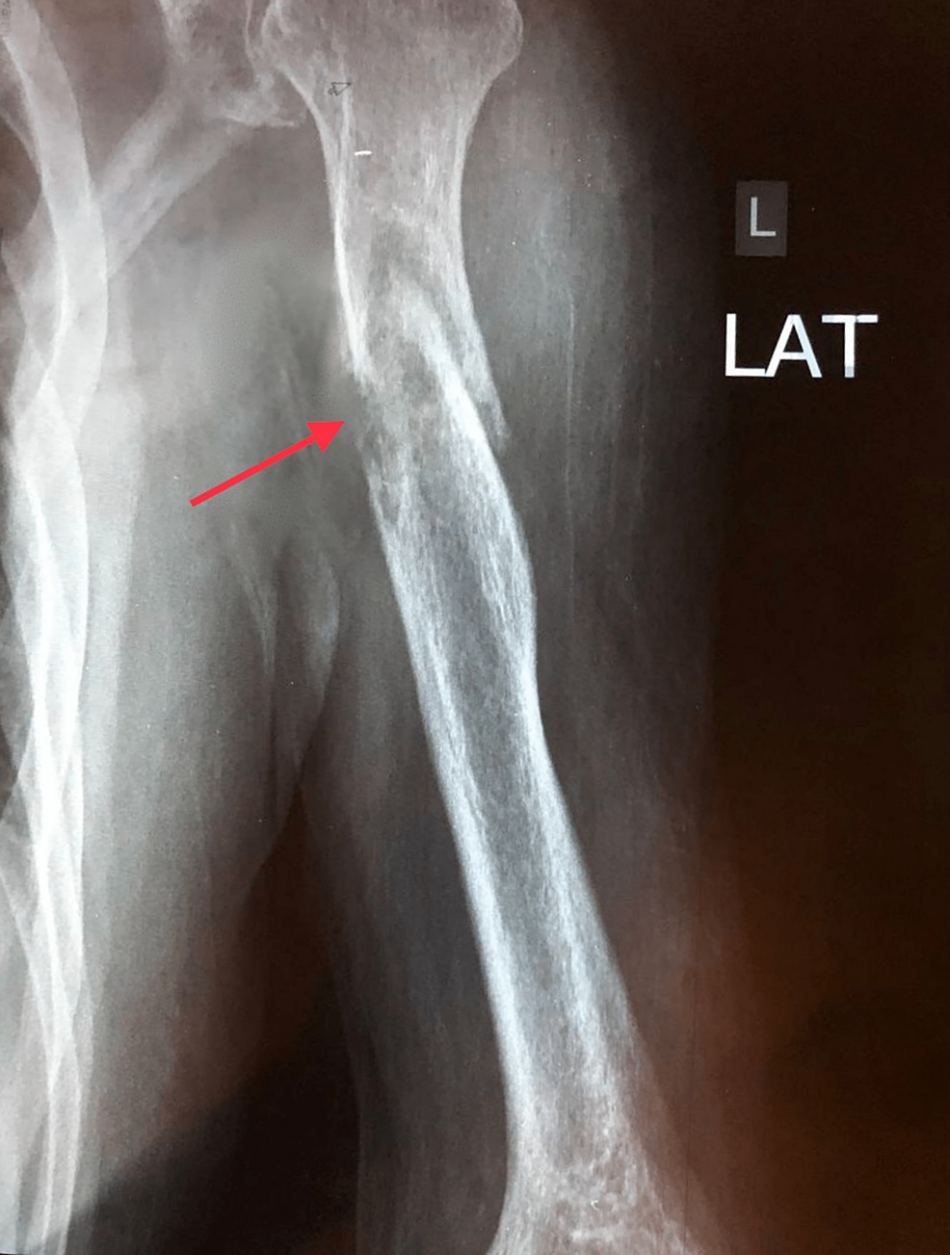
Plain radiograph showing pathological fracture in the left humeral shaft.

**Figure 4:**
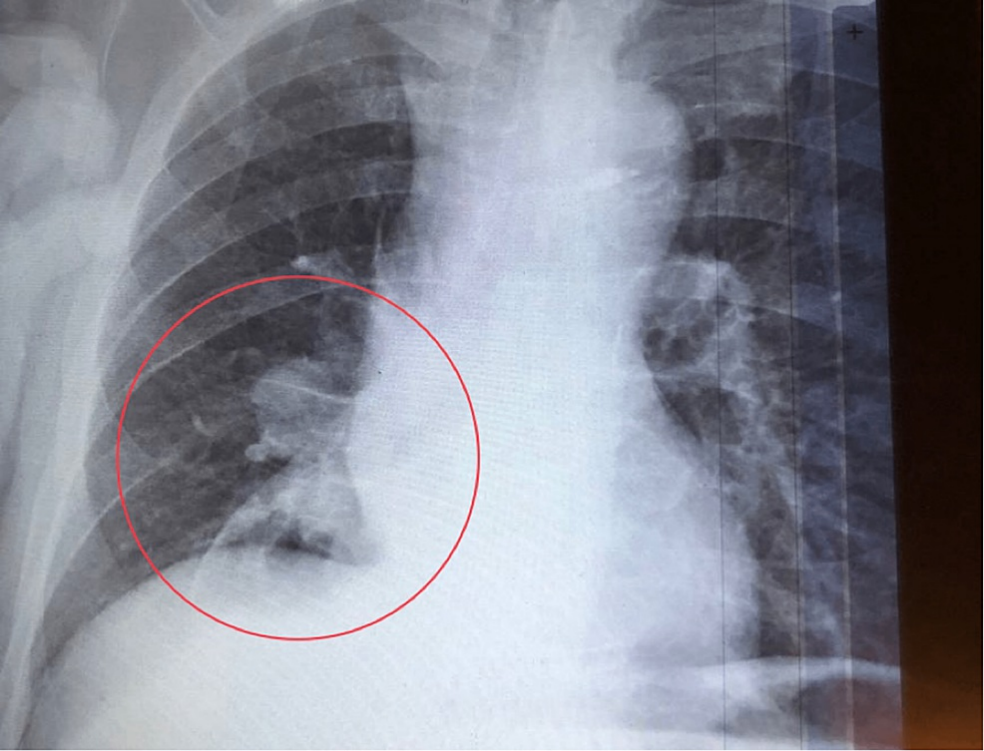
Plain chest radiograph showing right lower lobe lung lesion.

**Figure 5:**
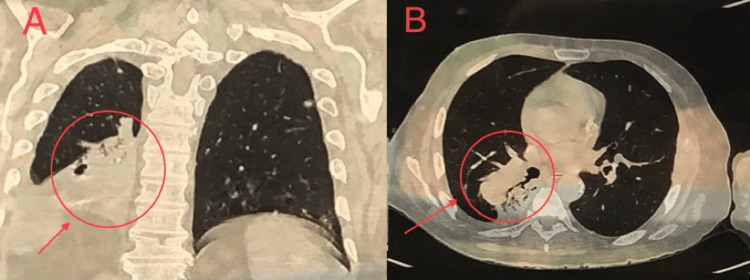
Contrast enhanced CT scan of the chest showing right lower lobe partly spiculated partly lobulated lung lesion in coronal section (A) & transverse section (B).

During the third week of follow up, he reported unbearable pain over the left arm. Plain radiography revealed a pathological fracture in the humerus (Figure 3). Secondary bone metastasis was suspected, and a simple plain chest radiography unexpectedly showed a suspicious right lower lobe lesion (Figure 4). Thus, a contrast enhanced CT scan for the chest, abdomen and pelvis was done which revealed a big (7.5 x 7.7 x 8 cm) partly lobulated partly spiculated outline mass lesion that was seen involving the superior segment of the lower lobe of the right lung (Figure 5).

The lesion was encasing the right lower lobe bronchus 3 cm distal to the carina, and it was heterogeneously enhanced after contrast. A diagnosis of right lower lobe bronchogenic carcinoma was made with pleural, bilateral adrenal glands, retroperitoneal and bone metastasis (T4 N3 M1C). Unfortunately, any invasive procedure to confirm the diagnosis was refused by the patient and his family including bronchoscopy and core needle biopsy. Therefore, the decision of determining the diagnosis was dependable on the contrast enhanced CT scan results.

A multidisciplinary team (oncologist, internist, orthopaedist and interventional radiologist) directed the patient for six courses of palliative radiotherapy plus dexamethasone and analgesics. Unfortunately, the patient died after 3 weeks of palliative therapy when he was admitted to the hospital with acute renal failure and septic shock.

## Discussion

The incidence of advanced malignant tumors with bone metastasis is 25-70%; prostate and breast cancer are responsible for the majority of the skeletal metastases (up to 70%) while lung cancer represents only 30-40% [2,13]. In this case, the presentation of lower backache with significant unexplained weight loss in an elderly patient had raised the suspicion of a hidden malignant tumour [5].

Secondary bone tumours (metastasis from primary malignancy) carried a high possibility in this patient since the primary bone malignancy, per se, is rare [1]. The normal PSA level and digital rectal examination (DRE), besides the unremarkable ultrasonography of the pelvis, had made the prostate cancer less likely to be the primary malignancy. Although the patient and his family refused bone marrow aspiration and biopsy, multiple myeloma did not fulfill the diagnostic criteria despite the presence of osteolytic spine lesion with normocytic normochromic anaemia.

On the other hand, colorectal cancer was still a possibility in such a presentation of an anaemic patient with unintended weight loss, besides a significant family history of colorectal cancer. Therefore, colonoscopy was done, but it didn’t reveal any abnormalities and this result reflects the fact that skeletal metastasis is a rare event in primary colorectal carcinomas [14,15]. Hence, the suspicion of primary colorectal cancer had faded, especially when CEA level and DRE were both normal.

Although the patient was a non-smoker and there was no specific sign or symptom of a primary lung pathology, a simple plain chest radiograph unexpectedly showed a suspicious right lower lobe lesion; therefore, a contrast-enhanced CT scan for the chest, abdomen, and pelvis was done which revealed a bronchogenic carcinoma in the right lower lobe of the lung. Such a rare presentation in the absence of any clinical sign of underlying lung disease was reported in only very few cases in the literature [9,10,11,12]. However, all of them presented with either skull or carpal bone metastasis, unlike our case.

The patient was treated with palliative radiotherapy plus dexamethasone and analgesics as the treatment options in this stage of advanced bronchogenic carcinoma (T4 N3 M1C) should focus on palliative therapy [16]. Despite that, he died after 3 weeks of treatment. The prognosis of malignant tumors with bone metastasis is extremely poor [17,18]. Lung cancer had the lowest 1-year survival rate (10%) among solid tumours after bone metastasis [19].

The significant family history of malignancy may indicate a pattern of underlying genetic mutation that requires further investigations. Patients with Lynch syndrome, which is also known as hereditary non-polyposis colorectal cancer, are at high risk of developing colorectal and endometrial carcinomas among other malignant tumours; however, the risk of developing lung cancer is considered the same as the general population [20].

## Conclusions

Bone metastases in lung cancer predict a poor prognosis and short-term survival. This case highlights a rare presentation of a hidden bronchogenic carcinoma which was initially presented with bone metastasis to the spine and humerus in the absence of any specific sign or symptom of underlying lung pathology, except for an unexpected radiological finding of a suspicious lung lesion.

The diagnosis of such a challenging presentation requires a high index of suspicion as it can be easily confused with multiple myeloma or prostate cancer being the primary tumour. If the patient had been sent for plain chest radiography at first, lots of time and effort could be saved in reaching the diagnosis without the need for further sophisticated or invasive diagnostic procedures.
